# New Species of Bioluminescent *Mycena* Sect. *Calodontes* (Agaricales, Mycenaceae) from Mexico

**DOI:** 10.3390/jof9090902

**Published:** 2023-09-01

**Authors:** Alonso Cortés-Pérez, Laura Guzmán-Dávalos, Virginia Ramírez-Cruz, Alma Rosa Villalobos-Arámbula, Eduardo Ruiz-Sanchez, Florencia Ramírez-Guillén

**Affiliations:** 1 Departamento de Botánica y Zoología, Universidad de Guadalajara, Zapopan 45200, Jalisco, Mexico; alonso.cortes5231@alumnos.udg.mx (A.C.-P.); eduardo.ruiz@academicos.udg.mx (E.R.-S.); 2 Departamento de Biología Celular y Molecular, Universidad de Guadalajara, Zapopan 45200, Jalisco, Mexico; avillal@cucba.udg.mx; 3 Instituto de Ecología, A.C., Xalapa 91073, Veracruz, Mexico; florencia.ramirez@inecol.mx

**Keywords:** agarics, cloud forest, luminescent, mycenoid, phylogeny, taxonomy

## Abstract

*Mycena* section *Calodontes* is macromorphologically distinguished by the collybioid or mycenoid basidiome, which is pink, purple, or violet, and, rarely, reddish-brown or yellowish. It is further characterized by the presence of oxalate crystals in the basal mycelium. The section comprises approximately 40 taxa, of which only five species and one variety exhibit bioluminescence. As part of an extensive study on *Mycena* sect. *Calodontes* in Mexico, specimens belonging to this section were collected and subjected to morphological analysis. Sequences from the nuclear internal transcribed spacer (ITS) of nuclear ribosomal DNA, RNA polymerase II large subunit Rpb1 (*rpb1*), and translation elongation factor-1α (*Tef-1α*) were generated to infer the relationships within *Mycena* sect. *Calodontes* using maximum likelihood and Bayesian inference. The phylogenetic evidence, along with the macro- and micromorphological features, supported the recognition of five new bioluminescent species within *Mycena* sect. *Calodontes.* Detailed macro- and micromorphological descriptions, line-drawing illustrations, and light and dark photographs of the new species are provided.

## 1. Introduction

Bioluminescent fungi are widely distributed across the world and are found in tropical and temperate forests. Currently, approximately 103 species are known, with the majority distributed in Australia, Brazil, China, India, Japan, and Malaysia [1,2,3,4,5,6,7]. Bioluminescent mushrooms belong to the phylum Basidiomycota, within the order Agaricales, and are grouped into four lineages: (1) *Armillaria* (Fr.) Staude (Physalacraceae), (2) Mycenaceae, (3) a group consisting of *Omphalotus* Fayod and *Neonothopanus* R.H. Petersen & Krisai (Omphalotaceae), and (4) the Lucentipes clade (taxonomic placement uncertain) [2,3,8]. Mycenaceae comprises at least 65 bioluminescent species, which are classified under the genera *Favolaschia* (Pat.) Pat., *Filoboletus* Henn., *Mycena* (Pers.) Roussel, *Panellus* P. Karst., *Resinomycena* Redhead & Singer, and *Roridomyces* Rexer [2,4,5,9]. Among these 65, *Mycena* accounts for the largest number of bioluminescent taxa, approximately 40 species classified into 18 sections [3,10,11].

*Mycena* sect. *Calodontes* (Fr. ex Berk.) Quél. is one of the sections with bioluminescent taxa, with five species and one variety exhibiting this feature: *M*. *cahaya* A.L.C. Chew & Desjardin, *M*. *pura* (Pers.) P. Kumm., *M*. *rosea* Gramberg, *M*. *seminau* A.L.C. Chew & Desjardin, *M*. *sinar* A.L.C. Chew & Desjardin, and *M*. *sinar* var. *tangkaisinar* A.L.C. Chew & Desjardin [1,3].

*Mycena* sect. *Calodontes* comprises approximately 44 described species worldwide [1,12,13,14,15,16,17,18,19,20,21,22,23,24,25]. These species are characterized by collybioid or mycenoid basidiome, which can be pink, purple, violet, and, rarely, reddish-brown or yellowish. The pileus is glabrous, and the stipe possesses strigose mycelium at the base, along with oxalate crystals. The basidiospores are typically amyloid, although in some cases they can be inamyloid. The cystidia are prominent, fusiform to subcylindrical or clavate, with broadly rounded or rostrate apex, hyaline or pigmented content, and a raphanoid odor [15,21,23,26]. The section has been classified into four subsections based on micromorphological characters. The four subsections are *Generosae* Maas Geest. & de Meijer, which comprises species with amyloid basidiospores, cheilocystidia characterized by “narrowed necks that are not broadly rounded” at the apex, and the absence of pleurocystidia; *Marginatae* J.E. Lange, which comprises species with amyloid basidiospores, purple-brown cheilocystidia, and the presence of pleurocystidia; *Purae* (Konrad & Maubl.) Maas Geest., which includes species with amyloid basidiospores, clavate, fusiform, subcylindrical cheilocystidia that are apically rounded or somewhat attenuated, and hyaline pleurocystidia; and *Violacella* Singer ex Maas Geest., the species of which subsection have inamyloid basidiospores, clavate, subcylindrical, subfusiform cheilocystidia that are apically obtuse, or rarely mucronate, and absent pleurocystidia [1,21,23]. Phylogenetic studies have revealed that subsection *Purae* is polyphyletic [1,16,20,25]. Additionally, Harder et al. [17] concluded that the amyloid reaction of basidiospores is not a reliable diagnostic feature for distinguishing species within *M*. sect. *Calodontes*.

Furthermore, *M.* sect. *Calodontes* is among the sections that have been extensively studied in a phylogenetic context. Harder et al. [16] conducted a phylogenetic analysis using the rDNA ITS and found a correspondence between the recognized species based on morphology and the lineages obtained from their analysis, except for *M. pura* and *M*. *diosma* Krieglst. & Schwöbel. Subsequently, Harder et al. [18] identified 11 cryptic lineages within *M. pura*. In the studies of Olariaga et al. [25] and Li et al. [19,20], three nuclear DNA regions were used to describe new taxa.

In Mexico, 10 species of bioluminescent fungi are known, including *Panellus stipticus* (Bull.) P. Karst. and nine species belonging to the genus *Mycena* [27,28,29] (Table 1). On the other hand, only two species from *M.* sect. *Calodontes* have been recorded in the country, namely, *M*. *pearsoniana* Dennis ex Singer and *M*. *pura* s.l. The latter has been mentioned in the literature as bioluminescent [3,17,30]. However, this bioluminescent characteristic has not been described or observed in Mexican specimens. Additionally, Mexican specimens have not been included in the phylogenetic studies of *M*. sect. *Calodontes*.

During our investigations on *Mycena* in Mexico, we collected specimens exhibiting morphological characteristics similar to those of *M*. sect. *Calodontes*, such as pink, violet, or purple basidiomata. This led us to believe that some of the specimens might belong to the *M*. *pura* complex. Consequently, we anticipated that they could represent new taxa, given the limited attention the genus has received in Mexico. The aims of this study were (1) to make a phylogenetic study of the Mexican species of *M*. sect. *Calodontes*, and (2) to describe potentially five new bioluminescent species, based on the phylogenetic results as well as macro- and micromorphological features. Our intention was to contribute to the understanding of bioluminescent species within *Mycena*, focusing specifically on the *Calodontes* section.

## 2. Materials and Methods

### 2.1. Study Area

*Mycena* specimens were collected in two localities in Mexico, both with cloud forest. The first locality was Estación Científica Las Joyas, located in the Reserva de la Biósfera Sierra de Manantlán, Jalisco. This area covers 1257 ha and has an altitudinal range of 1500 to 2250 m a.s.l. The dominant tree species in this locality included *Clethra fragrans* L.M. González & R. Delgad., *Cornus disciflora* Moc. & Sessé ex DC., *Dendropanax arboreus* (L.) Decne. & Planch., *Magnolia iltisiana* A. Vázquez, and *Quercus* spp. [31]. The second locality was La Martinica, Banderilla, located in the central part of Veracruz. This area encompasses a Protected Natural Area, covering 52.36 ha with an elevation ranging from 1570 to 1650 m a.s.l. The dominant tree species observed at this site were *Carpinus tropicalis* (Donn. Sm.) Lundell, *Clethra mexicana* DC., *Liquidambar styraciflua* L., *Lippia myriocephala* Schltdl. & Cham., *Myrsine coriacea* (Sw.) R.Br., and *Quercus* spp. [32].

### 2.2. Morphological Study

The studied specimens were deposited in the fungal collection of the Herbarium of the Institute of Botany at the University of Guadalajara (IBUG), with isotypes also deposited at XAL (herbarium abbreviations according to Thiers [33]). The macroscopy terminology followed Maas Geesteranus [21], while microscopic features were based on Largent et al. [34] and Vellinga [35]. Color codes in the descriptions were referenced from Kornerup and Wanscher [36]. Free-hand sections were prepared from various parts of the dried basidiomata, which were first rehydrated in 70% ethyl alcohol, then mounted in a 5% solution of KOH, Melzer’s reagent (to determine amyloidity, inamyloidity, dextrinoidity, or non-dextrinoidity reactions), or Congo red. A minimum of 30 measurements were taken for each taxonomic structure. Basidiospore statistics included X_m_, the arithmetic mean of the spore length by spore width (±SD) for *n* spores measured for a given specimen; Q, the ratio of spore length to spore width, expressed as a range for all spores measured; and Q_m_, the mean of all Q values (±SD). Basidiospores, cystidia, and other structures were drawn using a drawing tube mounted on a Leica DME light microscope (Wetzlar, Germany). All measurements represented the minimum and maximum dimensions observed, with measurements indicating values considered outside the normal range in parentheses. Microscopic structures were photographed using Axio Vision 4 software on a Zeiss Primostar 3 optical microscope. Photographs capturing bioluminescence in complete darkness were taken with the camera of a Redmi Note 8 smartphone (Beijing, China) equipped with a wide lens (32 s exposure, ISO 3200, and 1.79 f).

### 2.3. Extraction, Amplification, and Sequencing

Genomic DNA was extracted from herbarium specimens using the method proposed by Aljanabi and Martinez [37], with some modifications. The ITS, *rpb1*, and *Tef-1α* were amplified through polymerase chain reaction (PCR). Each 53 µL PCR contained 50 µL of PCR mix (35 µL of PCR water, 6 µL of 10X Taq reaction buffer without MgCl_2_, 3.0 µL of 50 mM MgCl_2_, 3 µL of 5 mM dNTP, 3 µL of 2 µg/µL bovine serum albumin (BSA), 0.5 µL of each 10 µM primer, 0.15 µL of Platinum^TM^ Taq DNA Polymerase High Fidelity (5 U/µL), and 1 µL of DNA template. The ITS1F/ITS4 primer pairs were used to amplify the whole ITS, and in some cases ITS1F/ITS2 and ITS3/ITS4S were used to amplify fragments of the ITS region [38,39,40]. To amplify the *rpb1* gene, we used the primers Mp-f1/Mp-r1 [18], and to amplify the *Tef*-*1α* gene, the primer pairs used were EF1-983F/EF1-1567R and tEFMp-f2/Mp-r2 [18,41]. PCR amplifications were performed in an ESCO Swift MaxPro thermal cycler. PCR products were cleaned up using Illustra GFX columns (GE Healthcare (Chicago, IL, USA)). Purified products were sent to the University of Arizona Genetics Core for sequencing.

### 2.4. Edition, Alignment, and Phylogenetic Analysis

Sequence review and editing were performed using Chromas vs. 1.45 [42]. The matrix was constructed and aligned using PhyDE vs. 0.9971 [43]. The alignment was visually inspected and manual corrections were made when necessary. In addition, we downloaded 157 sequences available in GenBank that were generated in previous studies [1,14,16,17,18,19,20] (Table 2). *Mycena rubromarginata* (Fr.) P. Kumm. was used as an outgroup for phylogenetic analysis. Three separate data matrices were created for ITS, *rpb1*, and *Tef*-*1α*, which were analyzed individually using the program raxmlGUI vs. 2.0 [44] to ensure that there were no conflicting topologies. Subsequently, the matrices were concatenated, and maximum likelihood (ML) and Bayesian inference (BI) analyses were conducted. ML analyses were performed in raxmlGUI vs. 2.0 [44] using the GTRGAMMA and empirically based frequency model, with 1000 bootstrap (BS) replicates with all models free and parameters estimated by raxmlGUI vs. 2.0 [44,45]. For the Bayesian analysis, the most appropriate evolutionary model for each dataset was selected using jModelTest vs. 2.1.10 with the Akaike criterion corrected [46]. BI analyses were conducted in MrBayes vs. 3.2.7 [47]. Two independent runs were performed, each consisting of ten million generations with trees sampled every 100 generations. The standard deviation of the split frequencies was examined to confirm the convergence between the independent runs. The first 25% of the samples representing the burn phase were discarded, and posterior probabilities (PP) were calculated from a 50% majority consensus tree of the remaining trees. The resulting trees were visualized using FigTree vs. 1.4.1 [48].

## 3. Results

### 3.1. Phylogenetic Analysis

A total of 189 sequences, representing 24 taxa, were used for the phylogenetic reconstruction of *Mycena* sect. *Calodontes*. Of these, 32 sequences were generated in this study (12 ITS, 10 *rpb1*, and 10 *Tef*-*1α*). Detailed information for all sequences can be found in Table 2. The concatenated matrix comprised 70 terminals and 1281 positions, which were distributed as follows: ITS (1–529), *rpb1* exon (530–622), *rpb1* conserved intron (623–944), *Tef*–*1α* exons (945–1250), and *Tef*-*1α* conserved intron (1251–1281). Due to alignment ambiguities, the *Tef*-*1α* not conserved intron and 298 positions from the ITS region were removed. The substitution models for each partition were as follows: TrN+I+G for ITS, JC for *rpb1*, K80+G for *rpb1* intron, TVMef+I+G for *Tef*-*1α* exon, and GTR+G for *Tef*-*1α* intron. Phylogenetic analysis was performed on this concatenated dataset.

**Figure 1:**
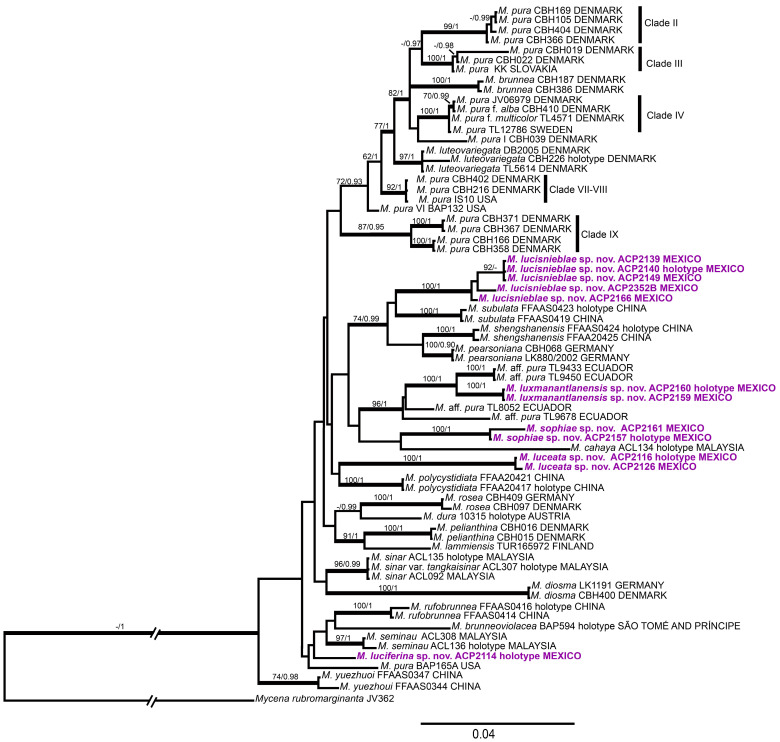
Phylogenetic tree of *Mycena* sect. *Calodontes* from the ML analysis of the ITS + *rpb1* + *Tef*-*1α* dataset. Maximum likelihood bootstrap values (≥70%) and posterior probabilities (≥95%) are shown on each branch. The hyphen (-) indicates that the value is absent. Branch lengths are scaled to the expected number of nucleotide substitutions per site. The new species are marked in purple.

Although ML and BI analyses generated slightly different topologies, the placement of the five Mexican taxa on both trees was consistent. The analysis identified 24 statistically supported clades (BS ≥ 70%, PP ≥ 0.90) that separated the different members of the *Mycena* sect. *Calodontes*. The ML tree (Figure 1) revealed that the *Mycena* collections from Mexico did not cluster with any clades of *M*. *pura* s.l. or known species of *M*. sect. *Calodontes*. Instead, the Mexican collections formed five monophyletic lineages, four of which had high statistical support: *M*. *luceata* (BS = 100%, PP = 1), *M*. *lucisnieblae* (BS = 100%, PP = 1), *M*. *luxmanantlanensis* (BS = 100%, PP = 1), and *M*. *sophiae* (BS = 100%, PP = 1). *Mycena lucisnieblae* was grouped in a statistically supported clade (BS = 74%, PP = 0.99), which included *M*. *shengshanensis* Z.W. Liu, Y.P. Ge & Q. Na and *M*. *subulata* Z.W. Liu, Y.P. Ge & Q. Na, described in China, as well as *M*. *pearsoniana*, known from North America and Europe.

*Mycena luxmanantlanensis* showed a sister relationship with a specimen from Ecuador, tentatively identified as *M*. aff. *pura* (BS = 100%, PP = 1). They formed a clade (BS = 96%, PP = 1) with other specimens from Ecuador, also identified as *M*. aff. *pura*. *Mycena sophiae* formed a clade with *M*. *cahaya* from Malaysia, although this relationship lacked statistical support. Furthermore, *M*. *luceata* and *M. polycystidiata* Z.W. Liu, Y.P. Ge, L. Zou & Q. Na from China were found to be sister species, but in an unsupported clade.

For the fifth new Mexican species, *M. luciferina*, we only had a DNA sequence from the single collected specimen. This sequence was positioned at the base of an unsupported clade that included *M. brunneoviolacea* A.C. Cooper, Desjardin & B.A. Perry from the Republic of São Tomé and Príncipe, *M. rufobrunnea* Z.W. Liu, Y.P. Ge & Q. Na from China, and *M. seminau* A.L.C. Chew & Desjardin from Malaysia.

### 3.2. Taxonomy

**Figure 2:**
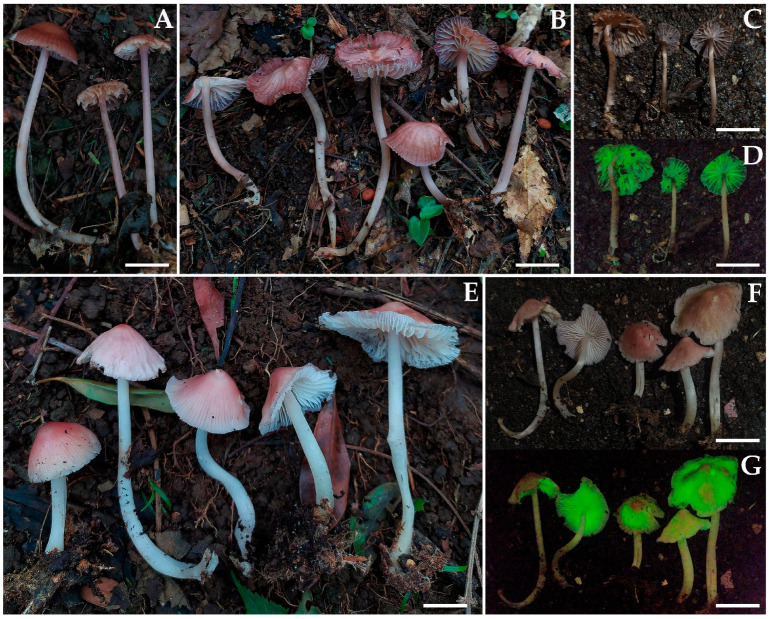
Basidiomes of bioluminescent species of *Mycena* sect. *Calodontes* from Mexico, in photographs with light and dark exposures. (**A**–**D**). *Mycena luceata* [(**A**). A. Cortés-Pérez 2115; (**B**). holotype A. Cortés-Pérez 2116; (**C**,**D**). A. Cortés-Pérez 2126)] (**E**–**G**). *M*. *luciferina* (holotype A. Cortés-Pérez 2114). Bars: (**A**,**B**,**E**) = 20 mm; (**C**,**D**) = 25 mm; (**F**,**G**) = 50 mm.

**Figure 3:**
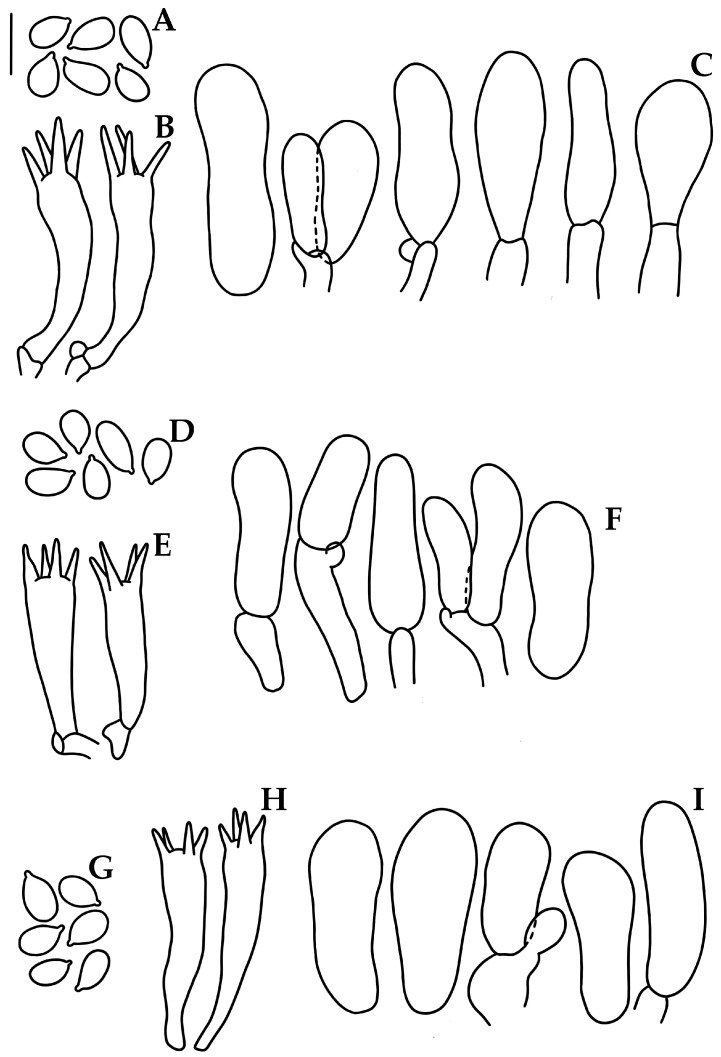
Microscopic features of *Mycena luceata*. (**A**,**D**,**G**). Basidiospores. (**B**,**E**,**H**). Basidia. (**C**,**F**,**I**). Cheilocystidia. (**A**–**C**). Holotype A. Cortés-Pérez 2116; (**D**–**F**). A. Cortés-Pérez 2126; (**G**–**I**). A. Cortés-Pérez 2115. Bar = 10 µm.

***Mycena luceata*** Cortés-Pérez, Guzm.-Dáv. & Ram.-Cruz, sp. nov. (Figure 2A–D and Figure 3).

MycoBank MB849395.

Etymology. From *luceata* (Latin), glow up, in reference to the brightly glowing hymenophore.

Holotype. MEXICO. VERACRUZ: Municipality of Banderilla, La Martinica, elev. 1639 m, 19° 35′ 13″ N, 96° 57′ 08″ W, gregarious, growing on leaf litter in cloud forest, 11 September 2021, *A. Cortés-Pérez 2116* (holotype IBUG, isotype XAL). GenBank: ITS OR233614, *rpb1* OR233746, *Tef1-α* OR233755.

Diagnosis. *Mycena luceata* is distinguished by the convex to plane-convex, hygrophanous, violet-brown to grayish-brown pileus; subdistant, intervenose, white to pinkish-white or orange-white lamellae; purplish-pink to pinkish-white stipe; 6–8 × 3.5–5 μm, Q = 1.2–2.1, amyloid basidiospores; 17–36 × 6–12 μm, narrowly to broadly clavate to cylindrical, obtuse, hyaline cheilocystidia, and bioluminescence only in the hymenophore.

Basidiomata medium size, collybioid. Pileus 20–43 mm diam., convex to convex-plane, sometimes with depressed center, margin translucent-striate to sulcate-striate, reflexed, even or eroded in age; surface glabrous, moist, hygrophanous, disc and striations or all violet-brown (10E8, 11E6), ruby (12E8), to grayish-violet (11E4) or pink (11A5), fading to pinkish-white (11A2) or grayish-brown (10D3). Context 1 mm thick, pinkish-white (11A2). Lamellae subdecurrent to adnate, subventricose, subdistant (14–20 reaching the stipe) with 1–3 series of lamellulae, intervenose, white or pinkish-white (11A2) to orange-white (6A2); edge even, pale. Stipe 38–82 × 2.5–5 mm, central or eccentric, cylindrical, with a swollen or narrow base, hollow; surface glabrous, apex purplish-pink (14A3) to purplish-white (14A2), pinkish-white (13A2) towards the base, with the base yellow-white (2A2); with white strigose basal mycelium. Odor not recorded. Bioluminescence in hymenophore, emitting bright green light.

Basidiospores (5.5–)6–8(–8.2) × 3.5–5(–5.5) μm (X_m_ = 6.6 ± 0.5 × 4.2 ± 0.4 µm, Q = 1.2–2.1, Q_m_ = 1.5 ± 0.1, *n* = 54), ellipsoid to elongated, thin-walled, hyaline, amyloid. Basidia 28–30 × 5–7 μm, clavate, 4-spored, with sterigmata 3–6 μm long, hyaline, inamyloid. Lamellar edge sterile. Cheilocystidia 17–36 (–37) × 6–12 μm, narrowly to broadly clavate to cylindrical, apex obtuse, thin-walled, hyaline. Pleurocystidia absent. Subhymenium ramose, non-gelatinous, hyaline, non-dextrinoid. Hymenophoral trama regular; hyphae 2–24 μm diam., cylindrical to inflated, thin-walled, non-gelatinous, hyaline, dextrinoid. Pileipellis an ixocutis 6.5–14 μm thick, hyphae 2–6 μm diam., thin-walled, hyaline, non-dextrinoid. Subcutis thick, hyaline, dextrinoid. Pileus trama hyphae 2–24 μm diam., interwoven, cylindrical to inflated, thin-walled, hyaline, dextrinoid. Stipitipellis hyphae 2–6 μm diam., thin-walled, non-gelatinous, hyaline, non-dextrinoid; medullary hyphae of the stipe 3–30 μm diam., thin-walled or wall up to 1 μm thick, hyaline, dextrinoid. Caulocystidia absent. Clamp connections present.

Habitat and distribution—Gregarious, growing on leaf litter in cloud forest, Mexico (Veracruz).

Additional specimens examined—Mexico, Veracruz, Municipality of Banderilla, La Martinica, 19° 35′ 13″ N, 96° 57′ 08″ W, elev. 1639 m a.s.l., 15 September 2021, *A. Cortés-Pérez 2115, 2126* (IBUG). Notes—*Mycena luceata* is distinguished by the violet-brown pileus, white lamellae, basidiospores averaging 6.6 × 4.2 μm, 17–36 × 6–12 μm, clavate to cylindrical cheilocystidia, and by the pileipellis as an ixocutis. A morphologically similar species is *M. fenestrata* Maas Geest. & de Meijer, described in Brazil, which has a purplish-brown to greyish-violet pileus, 6.7–8.1 × 3.6–4.5 μm basidiospores, and 18–35 × 8–15 μm, clavate, fusiform or subcylindrical cheilocystidia, but they are separate because *M. fenestrata* presents greyish-violet lamellae, dark purplish-brown stipe, and non-gelatinous pileipellis, and because of the presence on the stipe of 24–27 × 6.5–8 μm clavate terminal cells [23]. Another similar species is *M. diosma*, which has a basidiome with a dark violet-brown or reddish-purple hygrophanous pileus, but it is distinguished by the fact that *M*. *diosma* has dark brownish-violet to dark violet lamellae, larger cheilocystidia, 20–60(–80) × 3.5–20 μm, and pleurocystidia present [13,21]. *Mycena luceata* is distinguished from Malaysian bioluminescent species by macro- and micromorphological characters [1].

In the phylogenetic reconstruction, *M. luceata* was recovered as a possible sister to *M*. *polycystidiata* from China, but they are different because the Chinese species has a grayish-rose pileus with brownish-orange umbo, 50–78 × 14–31 μm utriform or subclavate cheilocystidia, abundant pleurocystidia, and caulocystidia present [20].

**Figure 4:**
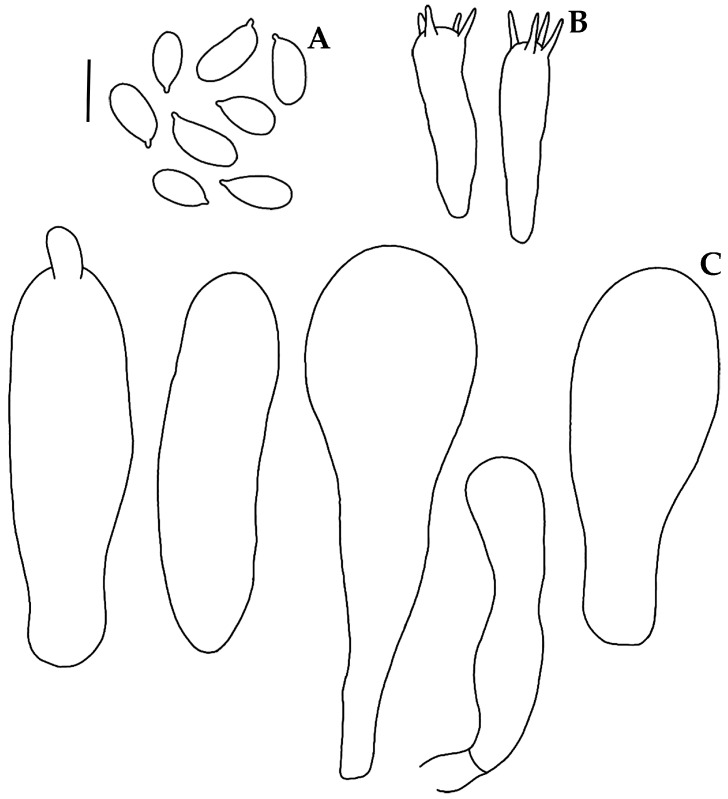
Microscopic features of *Mycena luciferina*. (**A**). Basidiospores. (**B**). Basidia. (**C**). Cheilocystidia. (**A**–**C**) Holotype A. Cortés-Pérez 2114. Bar = 10 µm.

***Mycena luciferina*** Cortés-Pérez, Guzm.-Dáv. & Ram.-Cruz, sp. nov. (Figure 2E–G and Figure 4).

MycoBank MB849396.

Etymology. From *lux* (Latin), light, -*ferre* (Latin), bringer: the bringer of light, in reference to the bioluminescent pileus.

Holotype. MEXICO. VERACRUZ: Municipality of Banderilla, 19° 35′ 18″ N, 96° 57′ 22″ W, elev. 1639 m a.s.l., gregarious, growing on leaf litter in cloud forest, 11 September 2021, *A. Cortés-Pérez 2114* (holotype IBUG, isotype XAL). GenBank: ITS OR233612, *rpb1* OR233744.

Diagnosis. *Mycena luciferina* is distinguished by the campanulate, reddish-pink, hygrophanous pileus; close, intervenose, white lamellae; white stipe; 6.4–8 × 3.6–4 μm, Q = 1.5–2.2, amyloid basidiospores; 33–64 × 10–17 μm, narrowly to broadly clavate to cylindrical, apex obtuse, hyaline cheilocystidia; with pleurocystidia, rare, similar to cheilocystidia; pileipellis an ixocutis, and bioluminescent pileus.

Basidiomata medium size, mycenoid. Pileus 32–47 mm diam., campanulate, occasionally umbonate; margin translucent-striate to sulcate-striate, even or eroded in age; surface glabrous, moist, hygrophanous, disc reddish-pink (11A7), grayish-red (11C6) to pink (11A5), elsewhere pale pink (11A2–3), with a white margin, fading pinkish-white (11A2). Context 0.5–1 mm thick, pinkish-white (11A2). Lamellae adnate, subventricose, close (26–31 reaching the stipe), with 2–3 series of lamellulae, intervenose, white to yellowish-white (3A2); edge even, concolorous. Stipe 45–82 × 4–5 mm, central or eccentric, cylindrical, with a narrow base, hollow; surface glabrous, white to towards the base yellowish-white (3A2); with white strigose basal mycelium. Odor not recorded. Bioluminescence in pileus, emitting bright green light.

Basidiospores (6–)6.4–8(–8.4)(–8.8) × (3.2–)3.6–4 μm (X_m_ = 7.4 ± 0.8 × 3.8 ± 0.2 µm, Q = 1.5–2.2, Q_m_ = 1.9 ± 0.1, *n* = 30), ellipsoid to elongated, thin-walled, hyaline, amyloid. Basidia 15.2–27.2 × 4.5–5.6 μm, clavate, 4-spored, with sterigmata 3–5 μm long, hyaline. Lamellae edge sterile. Cheilocystidia 33–64(–68) × (7–)10–17(–20) μm, narrowly to broadly clavate to cylindrical, apex obtuse, thin-walled, hyaline. Pleurocystidia rare, when present near the edge of the lamella, similar to cheilocystidia. Subhymenium ramose to subcellular, hyaline, non-dextrinoid, non-gelatinous. Hymenophoral trama regular, hyphae 2–20 μm diam., cylindrical to inflated, thin-walled, hyaline, dextrinoid, non-gelatinous. Pileipellis an ixocutis 25–35 μm thick, hyphae 1.5–6.5 μm diam., hyaline, non-dextrinoid. Subcutis with hyphae 2–39 µm diam., inflated or cylindrical, thin-walled, hyaline, dextrinoid. Pileus trama hyphae 2.5–22 μm diam., cylindrical to inflated, thin-walled, interwoven, hyaline, dextrinoid. Stipitipellis with hyphae 2–10 μm diam., thin-walled, non-gelatinous, hyaline, non-dextrinoid; medullary hyphae 2–26 μm diam., thin-walled, hyaline, dextrinoid. Clamp connections present.

Habitat and distribution—Gregarious, growing on leaf litter in cloud forest. Known from Mexico (Veracruz).

Notes—*Mycena luciferina* is characterized by a campanulate, reddish-pink to pale pink pileus, white intervenose lamellae; basidiospores averaging 7.4 × 3.8 μm, amyloid; cheilocystidia clavate to cylindrical, with a rounded apex, pleurocystidia rare, similar to cheilocystidia, an ixocutis, with simple hyphae, and by its strongly bioluminescent pileus. A morphologically similar species is *M. rosea*, a European species that has basidiome with a pink pileus but is distinguished by pale pink to lilaceous pink lamellae, larger cheilocystidia, 30–90 × 9–36 μm, and caulocystidia present [13,21]. It is also like some of the species in the *Mycena pura* complex, especially those with pink pileus; however, they are easily distinguished by having larger cheilocystidia and pleurocystidia [13,21].

BLAST (https://blast.ncbi.nlm.nih.gov/Blast.cgi, accessed on 1 January 2023) sequence similarity searches were performed; comparison of the ITS sequence of the Mexican specimen (ACP2114) showed 96% similarity with sequences determined as *Mycena pura* from Canada and the USA, and 95% similarity with a sequence determined as *M*. *rosea* from Italy. In the phylogenetic tree, *M. luciferina* was recovered in an unsupported clade formed by *M. brunneoviolacea* described from Africa [14], *M*. aff. *pura* clade X *sensu* Harder et al. from the USA [18], *M*. *rufobrunnea* from China [20], and *M. seminau* from Malaysia [1]. Although the Mexican specimen is within this clade, it is in an independent lineage, which can be distinguished from the rest of the taxa by its morphological characteristics. *Mycena brunneoviolacea* is distinguished by its dark violet-brown or dark brown pileus, narrowly lageniform to lageniform cheilocystidia and pleurocystidia, and caulocystidia present [14]. *Mycena rufobrunnea* is separated by the dark brown to reddish-brown or greyish-brown pileus and utriform cheilocystidia, sometimes clavate, and caulocystidia present [20]. *Mycena seminau* differs in that it has a dark brown to brown pileus, yellowish-gray to reddish-gray lamellae, and narrower cheilocystidia, 32.8–56.8 × 5.6–9.6 μm [1].

**Figure 5:**
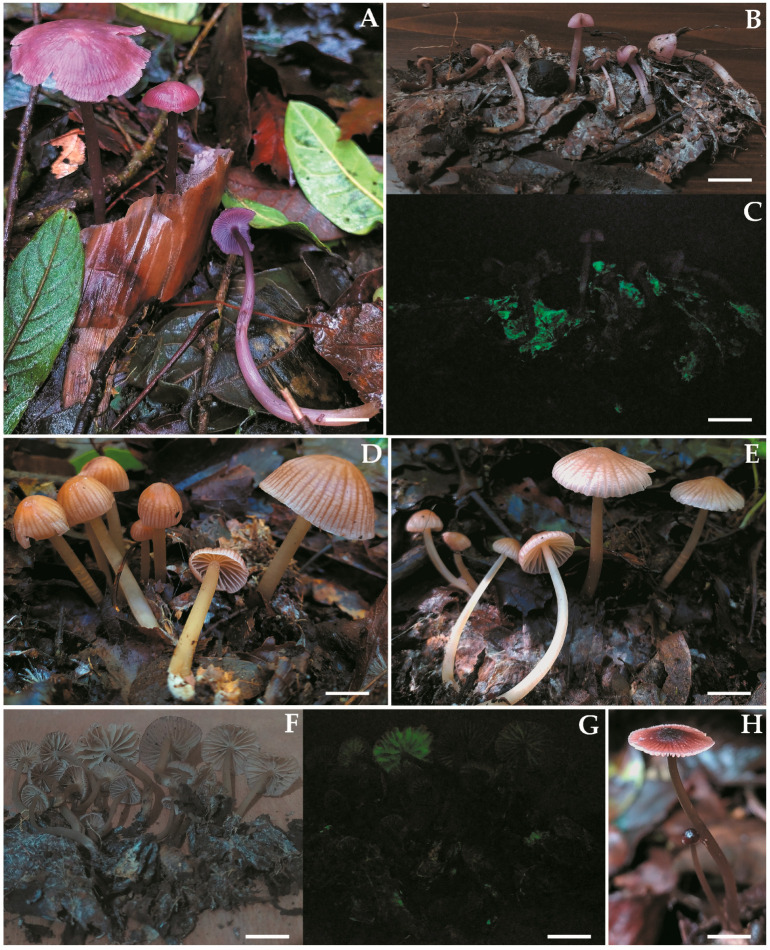
Basidiomes of bioluminescent species of *Mycena* sect. *Calodontes* from Mexico, in photographs with light and dark exposures. (**A**–**C**). *M*. *lucisnieblae* [(**A**). A. Cortés-Pérez 2139; (**B**,**C**). A. Cortés-Pérez 2149)]. (**D**–**G**). *M*. *luxmanantlanensis* [(**D**,**F**,**G**). holotype A. Cortés-Pérez 2160; (**E**). A. Cortés-Pérez 2159)]. (**H**). *M*. *sophiae* (holotype A. Cortés-Pérez 2157). Bars: (**A**,**F**,**G**) = 30 mm; (**B**,**C**) = 25 mm; (**D**,**E**) = 16 mm; (**H**) = 10 mm.

**Figure 6:**
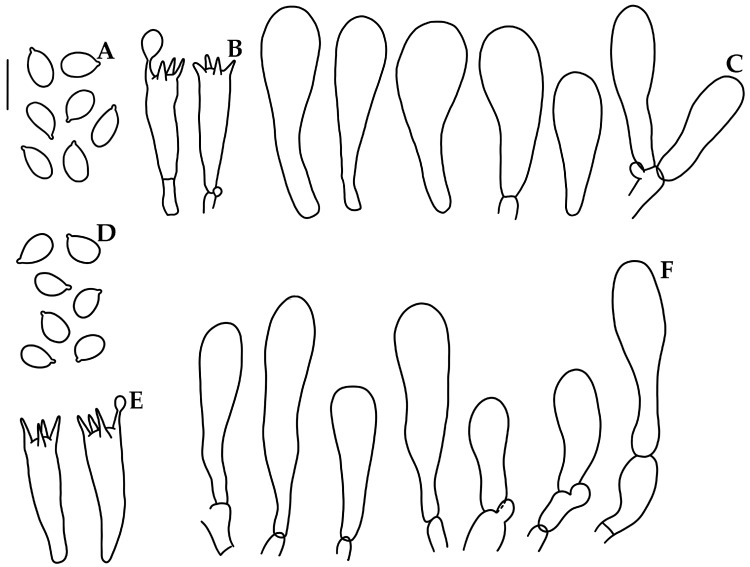
Microscopic features of *Mycena lucisnieblae*. (**A**,**D**). Basidiospores. (**B**,**E**). Basidia. (**C**,**F**). Cheilocystidia. (**A**–**C**) Holotype A. Cortés-Pérez 2140; (**D**–**F**) A. Cortés Pérez 2149. Bar = 10 µm.

***Mycena lucisnieblae*** Cortés-Pérez, Ram.-Cruz & Guzm.-Dáv., sp. nov. (Figure 5A–C and Figure 6).

MycoBank MB849397.

Etymology. From *lucis* (Latin), referring to light, -*niebla* (Spanish, from the Latin nebula), referring to the “bosque de niebla” or cloud forest, in reference to the bioluminescent mycelium that grows in the leaf litter of cloud forest.

Holotype. MEXICO. JALISCO: Municipality of Autlán de Navarro, Estación Científica Las Joyas, Sierra de Manantlán, 19° 35′ 15″ N, 104° 16′ 27″ W, elev. 1926 m a.s.l., gregarious, growing on leaf litter in cloud forest, 18 September 2021, *A. Cortés-Pérez 2140* (holotype IBUG, isotype XAL). GenBank: ITS OR233610, *rpb1* OR233742, *Tef1*-*α* OR233752.

Diagnosis. *Mycena lucisnieblae* is characterized by the hemispheric-convex to convex, purplish-pink or pink, hygrophanous pileus; arcuate-adnate, intervenose, violet-white to pale pink lamellae; pale violet to pinkish-white stipe; 5.5–7.4 × 4–5 μm, Q = 1.2–2, amyloid basidiospores; 24–41 × 8.5–13 μm, narrowly to broadly clavate, apex obtuse, hyaline cheilocystidia; pleurocystidia absent; pileipellis an ixocutis; without terminal hyphae in the pileipellis or caulocystidia, and bioluminescent mycelium.

Basidiomata small-sized, collybioid. Pileus 7–31 mm diam., hemispheric-convex to convex, some umbonate; margin smooth or translucent-striate to sulcate-striate, even or eroded in age; surface glabrous, moist, hygrophanous, disc and striations grayish-magenta (14D5–7) or purplish-pink (14A4), elsewhere pale pink (13A2), or all pink (13A2–3), fading pinkish-white (13A2). Context 0.2–0.8 mm thick, pinkish-white (13A2). Lamellae arcuate-adnate to adnate, narrow, close (22–29 reaching the stipe), with 2–3 series of lamellulae, intervenose, violet-white (16A2) to pale pink (13A2); edge even, pale. Stipe 26–78 × 1.5–4 mm, central or eccentric, cylindrical, with a swollen or narrow base, hollow; surface glabrous, pale violet (15A3) to violet-white (15A2) or purplish-white (14A2) to pinkish-white (10A2), with the base yellowish-white (3A2); with white strigose basal mycelium. Odor not recorded. Bioluminescence in mycelium, emitting green light, basidiomes non-luminescent.

Basidiospores 5.5–7.4(–8) × (3.8–)4–5 μm (X_m_ = 6.6 ± 0.5 × 4.4 ± 0.3 µm, Q = 1.2–2, Q_m_ = 1.4 ± 0.1, *n* = 53), ellipsoid, some elongated, thin-walled, hyaline, amyloid. Basidia 23–33 × 6–7 μm, clavate, 4-spored, with sterigmata 2–4 μm long, hyaline. Lamellae edge sterile. Cheilocystidia 24–41(–44)(–46) × (7.5–)8.5–13 (–14.5) μm, narrowly to broadly clavate, apex obtuse, thin-walled, hyaline. Pleurocystidia absent. Subhymenium subcellular to ramose, non-gelatinous, hyaline, non-dextrinoid. Hymenophoral trama regular; hyphae 2.5–12 μm diam., cylindrical to inflated, thin-walled, non-gelatinous, hyaline, dextrinoid. Pileipellis an ixocutis 13–15 μm thick, hyphae 2–5 μm diam., hyaline, non-dextrinoid. *Subcutis* with hyphae 3–19 µm diam., inflated or cylindrical, thin-walled or wall up to 0.8 μm thick, hyaline, dextrinoid. Pileus trama hyphae 2–31 μm diam., cylindrical to inflated, thin-walled, interwoven, hyaline, dextrinoid. Stipitipellis hyphae 2–5 μm diam., thin-walled, non-gelatinous, hyaline, non-dextrinoid; medullary hyphae of the stipe 2–21 μm diam., thin-walled or wall up to 0.8 μm thick, hyaline, dextrinoid. Clamp connections present.

Habitat and distribution—Gregarious, growing on leaf litter in cloud forest. Known from Mexico (Jalisco).

Additional specimens examined—Mexico, Jalisco, Municipality of Autlán de Navarro, Estación Científica Las Joyas, Sierra de Manantlán, 19° 35′ 15″ N, 104° 16′ 27″ W, elev. 1926 m a.s.l., 18 September 2021, *A. Cortés-Pérez 2139* (IBUG); 19 September 2021, *A. Cortés-Pérez 2149* (IBUG); 20 September 2021, *A. Cortés-Pérez 2166* (IBUG); 29 September 2022, *A. Cortés-Pérez 2352*-*B* (IBUG).

Notes—*Mycena lucisnieblae* is distinguished by its small basidiome, purplish-pink to pink pileus, violet-white to pale pink intervenose lamellae, pink-white stipe with white-yellow base, basidiospores averaging 6.6 × 4.4 µm, pleurocystidia absent, pileipellis an ixocutis, and by the bioluminescent mycelium that grows on leaf litter of tropical cloud forest trees. In the phylogenetic reconstruction, *M*. *lucisnieblae* was recovered in a clade formed by *M*. *pearsoniana, M*. *shengshanensis*, and *M*. *subulata*, in which it is distinguished by its morphological characteristics and by the habitat where it grows [17,20,21]. *Mycena pearsoniana* is a similar morphological species in the pileus with lilaceous or pink tinges; however, it is different by the vinaceous buff lamellae, “vinaceous buff or light cinnamon drab at apex and pale ochraceous buff at base” stipe, 5–6.3 × 3.5–3.8 μm basidiospores, and larger cheilocystidia, 30–80 × 7–12.5 μm, subfusiform, subcylindrical to clavate, with rounded apex or more rarely mucronate [17,21]. *Mycena shengshanensis* is distinguished by its brown to violet-brown pileus, white lamellae, moderately thick-walled clavate cheilocystidia, and by growing on the litter layer of *Larix gmelinii* (Rupr.) Kuzen [20]. *Mycena subulata*, described in China, is morphologically very similar and is the sister species of *M*. *lucisnieblae*; it is different in that *M. subulata* has 43–82 × 4–11 μm, narrowly fusiform with long neck, thick-walled cheilocystidia, clavate with tapering apices caulocystidia, and because it grows on the litter layer in mixed forests of *Pinus koraiensis* Siebold & Zucc., *Larix gmelinii*, and *Tila* sp. [20].

**Figure 7:**
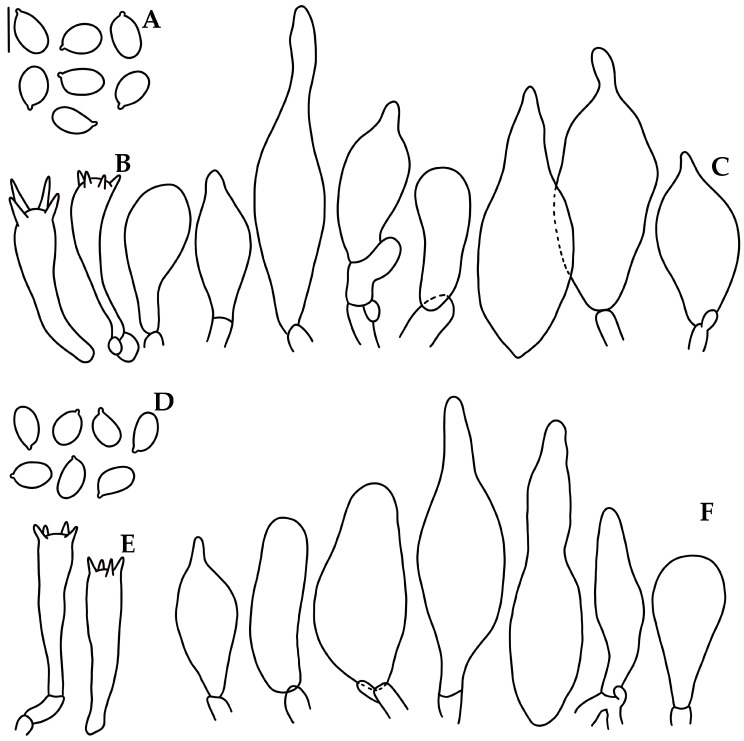
Microscopic features of *Mycena luxmanantlanensis*. (**A**,**D**). Basidiospores. (**B**,**E**). Basidia. (**C**,**F**). Cheilocystidia. (**A**–**C**) Holotype A. Cortés-Pérez 2160, (**D**,**E**). A. Cortés-Pérez 2159. Bar = 10 µm.

***Mycena* *luxmanantlanensis*** Cortés-Pérez, Ram.-Cruz & Guzm.-Dáv., sp. nov. (Figure 5D–G and Figure 7).

MycoBank MB849398.

Etymology. From *lux* (Latin), light, -*manantlanensis*, referring to the place Manantlán, in reference to the bioluminescent basidiomes that were collected in the Sierra de Manantlán.

Holotype. MEXICO. JALISCO: Municipality of Autlán de Navarro, Estación Científica Las Joyas, Sierra de Manantlán, 19° 35′ 15″ N, 104° 16′ 27″ W, elev. 1926 m a.s.l., gregarious, growing on leaf litter in cloud forest, 20 September 2021, *A. Cortés-Pérez 2160* (holotype IBUG, isotype XAL). GenBank: ITS OR233603, *rpb1* OR233737, *Tef1*-*α* OR233747.

Diagnosis. *Mycena luxmanantlanensis* is distinguished by the convex, tessellate pileus, brownish-orange to orange, with pink tinges, hygrophanous, then orange-white; distant to subdistant, adnate, intervenose lamellae; yellowish stipe; 6–8 × 4–5.2 μm, Q = 1.3–1.8, amyloid basidiospores; 25–50 × 8–17.5 μm, narrowly to broadly clavate hyaline cheilocystidia, with obtuse or rostrate apex; pileipellis an ixocutis; with bioluminescence in the mycelium and sometimes in the hymenophore.

Basidiomata medium size, collybioid. Pileus 9–55 mm diam., at first hemispheric-convex expanding to convex, some umbonate; translucent-tessellate and sulcate-striate, even to crenate or eroded in age; surface glabrous, moist, hygrophanous, disc and striations brownish-orange (5B8), grayish-orange (6B5), light orange or “melon” (5A6), elsewhere pale orange (5A3), orange-white (5A2), pink (7A2). Context 2–3 mm thick, fleshy, orangish-brown to white. Lamellae adnate, broad, distant to subdistant (13–18 reaching the stipe), with 2–3 series of lamellulae, intervenose, orange-white (6A2) to pinkish-white (7A2); edge even, concolorous. Stipe 44–78 × 2–6 mm, central or eccentric, cylindrical, with a swollen or narrow base, compressed at the base, hollow; surface glabrous, light yellow (3A4) to yellowish-white (3A2); with white (2A1) strigose basal mycelium. Odor not recorded. Bioluminescence in mycelium, sometimes in hymenophore, emitting bright yellowish-green light.

Basidiospores 6–8(–9) × (3.6–)4–5.2 μm (X_m_ = 6.9 ± 0.6 × 4.4 ± 0.4 µm, Q = 1.3–1.8, Q_m_ = 1.5 ± 0.1, *n* = 50), ellipsoid to elongated, thin-walled, hyaline, amyloid. Basidia 22–32 × 5–6.5 μm, clavate, 4-spored, with sterigmata 2–5 μm long, hyaline, inamyloid. Lamellar edge sterile. Cheilocystidia (20–)25–50(–52)(–56) × (7–)8–17.5 μm, narrowly to broadly clavate or subfusiform, apex obtuse or rostrate, thin-walled, hyaline. Pleurocystidia absent. Subhymenium ramose to subcellular, non-gelatinous, hyaline, non-dextrinoid. Hymenophoral trama regular, hyphae 2–26.5 μm diam., cylindrical to inflated, thin-walled, non-gelatinous, hyaline, dextrinoid. Pileipellis an ixocutis, 14.5–16 μm thick, hyphae 2–7 μm diam., hyaline, non-dextrinoid. Subcutis with hyphae 2.5–22.5 µm diam., inflated or cylindrical, thin-walled, hyaline, dextrinoid. Pileus trama hyphae 2.5–22.5 μm diam., cylindrical to inflated, interwoven, thin-walled, hyaline, dextrinoid. *Stipitipellis* hyphae 2–4 μm diam., thin-walled, non-gelatinous, hyaline, non-dextrinoid; medullary hyphae of the stipe 3–31 μm diam., hyaline, dextrinoid. Clamp connections present.

Habitat and distribution—Gregarious or scattered, growing on leaf litter in cloud forest, Mexico (Jalisco).

Additional specimens examined—Mexico, Jalisco, Municipality of Autlán de Navarro, Estación Científica Las Joyas, Sierra de Manantlán, 19° 35′ 15″ N, 104° 16′ 27″ W, elev. 1926 m a.s.l., 20 September 2021, *A. Cortés-Pérez 2159* (IBUG).

Notes—*Mycena luxmanantlanensis* is characterized by its orange pileus, strongly sulcate-striate, orange-white to pink-white, intervenose lamellae, and a yellow stipe, basidiospores averaging 6.9 × 4.4 µm, cheilocystidia clavate, fusiform with the apex obtuse or rostrate. A morphologically similar species is *M. luteovariegata* B. Harder & Læssøe described in Denmark, but the latter has a yellowish instead of orangish pileus, grayish-pink lamellae, reddish-gray stipe, and pleurocystidia [18]. *Mycena luxmanantlanensis* differs from the Malaysian bioluminescent species described by Chew et al. [1] in the following: *M*. *cahaya* has brown pileus, elongated basidiospores averaging 7.1 × 3.8 µm, and abundant clavate to ventricose cheilocystidia and pleurocystidia; *Mycena seminau* is different in that it has a dark brown to brown pileus, elongated to cylindrical basidiospores averaging 7.1 × 3.9 µm, and by the narrower cheilocystidia, measuring 32.8–56.8 × 5.6–9.6 µm [1]. Another similar bioluminescent species is *M*. *sinar*, which has a brownish-orange to yellowish-brown pileus, but differs in that it has subdecurrent yellowish-gray lamellae, basidiospores averaging 8.3 × 4 µm, and narrower cheilocystidia, 30.4–45.6 × 5.6–9.6 µm [1]. *Mycena sinar* var. *tangkaisinar* differs in the brown pileus, reddish-gray lamellae with pink margin, elongated basidiospores averaging 7.2 × 3.6 µm, cylindrical caulocystidia, and a luminescent stipe [1]. In the phylogenetic tree, *M*. *luxmanantlanensis* is sister to a taxon from Ecuador determined as *M*. aff. *pura* by Harder et al. [18]. These two species are of tropical affinity and in both the pleurocystidia are absent; however, Harder et al. [18] did not provide further morphological data on these Ecuadorian specimens, which could help to better understand the sister relationship between these two taxa.

**Figure 8:**
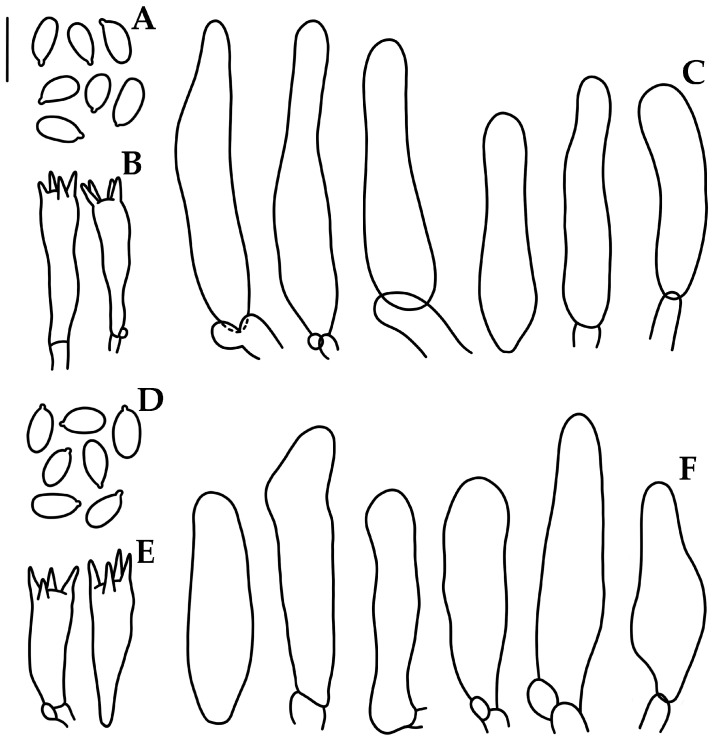
Microscopic features of *Mycena sophiae*. (**A**,**D**). Basidiospores. (**B**,**E**). Basidia. (**C**,**F**). Cheilocystidia. (**A**–**C**) Holotype A. Cortés-Pérez 2157. (**D**–**F**). A. Cortés-Pérez 2161. Bar = 10 µm.

***Mycena sophiae*** Cortés-Pérez sp. nov. (Figure 5H and Figure 8).

MycoBank MB849399.

Etymology. ‘*sophiae’* (Latin), dedicated to Sofía Cortés, niece of the first author.

Holotype. MEXICO. JALISCO: Municipality of Autlán de Navarro, Estación Científica Las Joyas, Sierra de Manantlán, 19° 35′ 15″ N, 104° 16′ 27″ W, elev. 1926 m a.s.l., gregarious, growing on leaf litter in cloud forest, 19 September 2021, *A. Cortés-Pérez 2157* (holotype IBUG, isotype XAL). GenBank: ITS OR233606, *rpb1* OR233739, *Tef1-α* OR233749.

Diagnosis. *Mycena sophiae* has a very small basidiome, with a convex to convex-plane, dark ruby to ruby pileus; adnate with a tooth, subdistant, pinkish-white lamellae; pinkish-white to greyish-orange stipe; 5.8–6.5 × 3–3.8 μm, Q = 1.4–2, amyloid basidiospores; 24–47 × 6.5–11 μm, cylindrical to narrowly clavate, obtuse cheilocystidia; a cutis as pileipellis; with bioluminescent mycelium.

Basidiomata small size, collybioid. Pileus 7–19 mm diam., hemispheric-convex to convex or convex-plane, margin translucent-striate to sulcate-striate, even or eroded in age; surface glabrous, moist, disc and striations dark ruby (12F8) to ruby (12D8), elsewhere pale or pastel red (9A3–4) to pinkish-white (8A2), margin white. Context thin, vinaceous pink to pinkish-white (11A2). Lamellae adnate with a tooth, narrow, subdistant (15–20 reaching the stipe), with 2–3 series of lamellulae, intervenose, pinkish-white (10A2); edge even, concolorous. Stipe 22–55 × 1–1.5 mm, central or eccentric, cylindrical, with a swollen base, hollow; surface glabrous, pinkish-white (11A2) to reddish-brown (8–9E5), with the base pale brownish-orange (7C4); with white strigose basal mycelium. Odor not recorded. Bioluminescence of mycelium observed but not documented, basidiomes non-luminescent.

Basidiospores (5.2–)5.8–6.5(–6.8) × 3–3.8 μm (X_m_ = 6.0 ±0.3 × 3.3 ±0.2 µm, Q = 1.4–2, Q_m_ = 1.8 ±0.1 *n* = 63), ellipsoid to elongated, thin-walled, hyaline, amyloid. Basidia 19–24 × 5–6 μm, clavate, 4–spored, with sterigmata 3–4 μm long, hyaline. Lamellar edge sterile. Cheilocystidia 24–47(–52) × 6.5–11 (–13) μm, cylindrical to narrowly clavate, obtuse, thin-walled, hyaline. Pleurocystidia absent. Subhymenium ramose, non-gelatinous, hyaline, non-dextrinoid. Hymenophoral trama regular, hyphae 2–24 μm diam., cylindrical to inflated, thin-walled, non-gelatinous, hyaline, dextrinoid. Pileipellis a cutis, hyphae 2–5 μm diam., hyaline, non-dextrinoid. Subcutis hyphae 2.6–33 µm diam., inflated or cylindrical, thin-walled or wall up to 1 μm thick, hyaline, dextrinoid. Pileus trama hyphae 3–22.6 μm diam., cylindrical to inflated, thin-walled, interwoven, hyaline, dextrinoid. Stipitipellis hyphae 1.5–10 μm diam., thin-walled, non-gelatinous, hyaline, non-dextrinoid; medullary hyphae of the stipe 2–25 μm diam., thin-walled or wall up to 1 μm thick, hyaline, dextrinoid. Clamp connections present.

Habitat and distribution—Gregarious, growing on leaf litter in cloud forest, Mexico (Jalisco).

Additional specimens examined—Mexico, Jalisco, Municipality of Autlán de Navarro, Estación Científica Las Joyas, Sierra de Manantlán 19° 35′ 15″ N, 104° 16′ 27″ W, elev. 1926 m a.s.l., 20 September 2021, *A. Cortés-Pérez 2161* (IBUG).

Notes—*Mycena sophiae* is distinguished by its very small basidiome, ruby pileus, pinkish-white lamellae, relatively small amyloid basidiospores, and cylindrical to narrowly clavate cheilocystidia. *Mycena sirayuktha* Aravind. & Manim., described in India [12], is a morphologically similar species in its greyish-red pileus and intervenose lamellae, but is different by the pleurocystidia present and gelatinized lageniform, fusiform, clavate, or utriform cheilocystidia. Other similar species are *M. clarkeana* Grgur. and *M*. *nullawarrensis* Grgur., described in Australia, which have a reddish-brown pileus, but both species are different by having pleurocystidia [15]. *Mycena cahaya* from Malaysia could be a sister group of *M. sophiae*; they are different because *M*. *cahaya* has a brown pileus, basidiospores averaging 7.1 × 3.8 μm, and ventricose to fusoid mucronate cheilocystidia, and pleurocystidia present [1]. *Mycena lucisnieblae* has small basidiomata resembling those of *M. sophiae* in size but is distinguished by the fact that *M. lucisnieblae* has pink or purplish-pink basidiomata.

## 4. Discussion

This study represents the first occurrence of bioluminescent species belonging to *Mycena* sect. *Calodontes* in Mexico. With the inclusion of these five species, the global count of bioluminescent fungi increases to 108, with 15 taxa recorded in that country. The Mexican bioluminescent species of *M.* sect. *Calodontes* are exclusively found in cloud forests and can be distinguished from species in other sections by their pileus color, which can be pink, red, purple, orange, or violet, their intervenose lamellae, the presence of oxalate crystals on the basal mycelium, the absence of pleurocystidia (except in *M*. *luciferina*), the absence of terminal hyphae in pileipellis and stipitipellis, the pileipellis an ixocutis (except in *M*. *sophiae*), and bioluminescence exhibited in the mycelium or some part of the basidiomata, or both.

According to the traditional classification, *M*. *luciferina*, characterized by its hyaline cheilocystidia and pleurocystidia, could be classified under *M.* sect. *Calodontes* subsect. *Purae*. *Mycena luxmanantlanensis,* with its fusiform cheilocystidia with rostrate apex, could be placed in the subsect. *Generosae*. Lastly, *M*. *luceata*, *M. lucisnieblae*, and *M*. *sophiae* could be grouped under subsect. *Violacella* due to their cheilocystidia with a rounded apex and the absence of pleurocystidia. However, the presence of amyloid basidiospores differentiates them from the species within subsect. *Violacella* [21,23]. As observed in other studies [1,18,20,25], these subsections are polyphyletic, and the micromorphological characters used in the infrageneric classification exhibit homoplasy. The presence of oxalate crystals on the basal mycelium in the Mexican species was confirmed, consistent with reports by Olariaga et al. and Clémençon for certain species of the *M.* sect. *Calodontes* from Europe [25,26]. The information presented here enhances our understanding of the distribution and ecological aspects of the members of *M.* sect. *Calodontes,* specifically in the cloud forest of Mexico.

The phylogenetic analysis conducted on *M.* sect. *Calodontes* aligns with previous studies [1,18,20,25]. Most of the lineages identified at the species level exhibited strong support, while shallower branches displayed weaker values, consistent with findings in other studies [20,25]. Importantly, this study marks the first inclusion of specimens from *M.* sect. *Calodontes* in Mexico, affirming the presence of five distinct clades among the Mexican samples. Additionally, the 11 clades proposed by Harder et al. [16,17,18] for *M. pura* were indeed recovered; however, they appeared in separate linages from the newly identified species.

We must emphasize that the relationships between certain taxa within *M*. sect. *Calodontes* has shifted with the inclusion of Mexican specimens. For instance, Liu et al. [20] recovered *M*. *polycystidiata* as a sister species to *M. diosma*, while in our study a clustering of *M*. *luceata* and *M*. *polycystidiata* was observed, albeit without support. Similarly, in Liu et al. [20], *M*. *pura* clade IX was linked with *M*. *cahaya*; however, our phylogeny uncovered an unsupported clade of *M*. *cahaya* and *M*. *sophiae*. Furthermore, in Harder et al.’s phylogenetic analysis [18], a clade featuring Ecuadorian taxa labeled as *M*. aff. *pura* was identified as the sister group of *M*. *pearsoniana*. Likewise, Liu et al. [20] found a clade composed of Ecuadorian taxa clustered with *M*. *pearsoniana*, *M*. *shengshanensis*, and *M*. *subulata* from China. In contrast, our analysis placed the Ecuadorian taxa together with *M*. *luxmanantlanensis* from Mexico in a well-supported clade. Additionally, another supported clade included *M*. *pearsoniana*, *M*. *shengshanensis*, and *M*. *subulata* along with *M*. *lucisnieblae*. Likewise, *M*. *luceata* and *M*. *luxmanantlanensis* clustered in a large unsupported clade, along with *M*. *lucisnieblae, M. pearsoniana*, *M. shengshanensis*, and *M. subulata*. This clade represents a mixture of species with temperate affinities, such as *M*. *pearsoniana*, and tropical ones, from Ecuador and Mexico, distinct from the *M*. *pura* complex.

Mexico is one of the countries with the greatest richness of bioluminescent fungi, with a total of 15 species, most of which are exclusive to the country and with only a few known also from other countries. In that country, *M.* sect. *Calodontes* represents the section, so far, with the largest number of species of bioluminescent fungi. It is expected that the knowledge of bioluminescent species will increase as other plant communities and states in the country are explored. On other hand, our phylogenetic reconstruction allowed us to recognize the phylogenetic position of the five new species from Mexico as independent of the *M. pura* complex. The inclusion of the Mexican specimens in the phylogenetic analyses changed the panorama, generating new relationship hypotheses within *M*. sect. *Calodontes*. In this sense, it is expected that, as more taxa are added to the studies, the phylogenetic relationships of this section will continue changing. Finally, this work is the basis for subsequent phylogenetic studies that include Mexican taxa.

## Figures and Tables

**Table 1:** Bioluminescent fungi in Mexico recorded in the literature.

Taxon	Section	Distribution	Bioluminescence	Reference
*Mycena fulgoris* Cortés-Pérez & Desjardin	*Rubromarginatae* Singer ex Maas Geest.	Mexico	Basidiome	[28]
*M. globulispora* Maas Geest. & de Meijer	*Supinae* Konrad & Maubl.	Brazil, Mexico	Basidiome	[28]
*M. guzmanii* Cortés-Pérez, Desjardin & B.A. Perry	*Euspeirea* (Berk. & Curt.) Sacc.	Mexico	Mycelium and basidiome	[28]
*M. lumina* Cortés-Pérez, Desjardin & A. Rockefeller	*Rubromarginatae*	Mexico	Mycelium and basidiome	[28]
*M. luxarboricola* Desjardin, B. A. Perry & Stevani	*Supinae*	Brazil, Mexico	Mycelium and basidiome	[29]
*M. luxfoliicola* Cortés-Pérez, Desjardin & Ram.-Cruz	*Nigrescentes* Maas Geest. & de Meijer	Mexico	Mycelium and basidiome	[28]
*M. nebula* Cortés-Pérez, Desjardin & A. Rockefeller	*Sanguinolentae* Maas Geest.	Mexico	Basidiome	[28]
*M. perlae* Cortés-Pérez, Desjardin & A. Rockefeller	*Amparoina* T. Bau & Q. Na	Mexico	Basidiome	[28]
*M. stylobates* (Pers.) P. Kumm.	*Basipedes* (Fr.) Quél.	Europe, Mexico, USA	Mycelium and basidiome	[27]
*Panellus stipticus* (Bull.) P. Karst.		Asia, Europe, North America	Mycelium and basidiome	[27]

**Table 2:** Specimens of *Mycena* employed in this study. Sequences generated for this work are in bold.

Species	Specimen Voucher	Country		GenBank Accession		References
			ITS	*rpb1*	Tef-1α	
*Mycena* aff. *pura*	TL8052	Ecuador	FN394623	KF723687	KF723641	[16,18]
*M.* aff. *pura*	TL9433	Ecuador	FN394622	KF723688	KF723642	[16,18]
*M.* aff. *pura*	TL9450	Ecuador	KJ144653	KF723689	KF723643	[16,18]
*M.* aff. *pura*	TL9678	Ecuador	FN394621	KF723690	KF723644	[16,18]
*M. brunneoviolacea*	BAP594, holotype	São Tomé and Príncipe	MH414546	–	–	[14]
*M. brunnea*	CBH187	Denmark	FN394564	KF723678	KF723632	[16,18]
*M. brunnea*	CBH386	Denmark	FN394565	KF723679	KF723633	[16,18]
*M. cahaya*	ACL134, holotype	Malaysia	KF537248	–	–	[1]
*M.* cf. *pura* I	CBH039	Denmark	FN394588	KF723680	KF723634	[16,18]
*M.* cf. *pura* II	CBH105	Denmark	FN394581	KF723671	KF723625	[16,18]
*M.* cf. *pura* II	CBH169	Denmark	FN394579	KF723672	KF723626	[16,18]
*M.* cf. *pura* II	CBH366	Denmark	FN394572	KF723673	KF723627	[16,18]
*M.* cf. *pura* II	CBH404	Denmark	FN394566	KF723674	KF723628	[16,18]
*M*. cf. *pura* III	CBH019	Denmark	FN394605	KF723675	KF723629	[16,18]
*M.* cf. *pura* III	CBH022	Denmark	FN394574	KF723676	KF723630	[16,18]
*M.* cf. *pura* III	KK	Slovakia	FN394606	KF723677	KF723631	[16,18]
*M.* cf. *pura* IV	CBH410	Denmark	FN394595	KF723667	KF723621	[16,18]
*M.* cf. *pura* IV	JV06979	Denmark	FN394585	KF723668	KF723622	[16,18]
*M.* cf. *pura* IV	TL4571	Denmark	FN394583	KF723669	KF723623	[16,18]
*M.* cf. *pura* IV	TL12786	Sweden	FN394591	KF723670	KF723624	[16,18]
*M.* cf. *pura* VI	BAP132	USA	FN394561	KF723660	KF723614	[16,18]
*M.* cf. *pura* VII	IS10/11/200	USA	FN394611	–	–	[16,18]
*M.* cf. *pura* VIII	CBH216	Denmark	FN394598	KF723662	KF723616	[16,18]
*M.* cf. *pura* VIII	CBH402	Denmark	FN394599	KF723663	KF723617	[16,18]
*M.* cf. *pura* IX	CBH166	Denmark	FN394607	KF723701	KF723655	[16,18]
*M.* cf. *pura* IX	CBH358	Denmark	FN394608	KF723702	KF723656	[16,18]
*M.* cf. *pura* IX	CBH367	Denmark	KF913022	KF723703	KF723657	[16,18]
*M.* cf. pura IX	CBH371	Denmark	KF913023	KF723704	KF723658	[16,18]
*M.* cf. *pura* X	BAP165A	USA	FN394563	KF723698	KF723652	[16,18]
*M. diosma*	CBH400	Denmark	FN394617	KF723699	KF723653	[16,18]
*M. diosma*	LK1191/2000	Germany	FN394619 *	KF723700	KF723654	[16,18]
*M. dura*	10315, holotype	Austria	FN394560	KF723694	KF723648	[16,18]
*M. lammiensis*	TUR165927	Finland	FN394552	KF723697	KF723651	[16,18]
***M. luceata***	**ACP2116, holotype**	**Mexico**	**OR233614**	**OR233746**	**OR233755**	**This study**
***M. luceata***	**ACP2126**	**Mexico**	**OR233613**	**OR233745**	**OR233754**	**This study**
***M. luciferina***	**ACP2114, holotype**	**Mexico**	**OR233612**	**OR23374** **4**	**–**	**This study**
***M. lucisnieblae***	**ACP2140, holotype**	**Mexico**	**OR233610**	**OR233742**	**OR233752**	**This study**
***M. lucisnieblae***	**ACP2139**	**Mexico**	**OR233611**	**OR233743**	**OR233753**	**This study**
***M. lucisnieblae***	**ACP2149**	**Mexico**	**OR233609**	**OR233741**	**OR233751**	**This study**
***M. lucisnieblae***	**ACP2166**	**Mexico**	**OR233607**	**OR233740**	**–**	**This study**
***M. lucisnieblae***	**ACP2352-B**	**Mexico**	**OR233608**	**–**	**OR233756**	**This study**
*M. luteovariegata*	CBH226, holotype	Denmark	FN394604	KF723664	KF723618	[16,18]
*M. luteovariegata*	TL5614	Denmark	FN394602	KF723666	KF723620	[16,18]
*M. luteovariegata*	DB2005/152	Denmark	FN394603	–	–	[16,18]
***M. luxmanantlanensis***	**ACP2160, holotype**	**Mexico**	**OR233603**	**OR233737**	**OR233747**	**This study**
***M. luxmanantlanensis***	**ACP2159**	**Mexico**	**OR233604**	**OR233738**	**OR233748**	**This study**
*M. pearsoniana*	CBH068	Germany	FN394614	KF723691	KF723645	[16,18]
*M. pearsoniana*	LK880/2002	Germany	FN394613	KF723693	KF723647	[16,18]
*M. pelianthina*	CBH015	Denmark	FN394549	KF723695	KF723649	[16,18]
*M. pelianthina*	CBH016	Denmark	FN394547	KF723696	KF723650	[16,18]
*M. polycystidiata*	FFAAS0417, holotype	China	ON427731	ON468456	ON468469	[20]
*M. polycystidiata*	FFAAS0421	China	ON427733	ON468458	ON468471	[20]
*M. rosea*	CBH097	Denmark	FN394556	KF723681	KF723635	[16,18]
*M. rosea*	CBH409	Germany	FN394551	KF723683	KF723637	[16,18]
*M. rufobrunnea*	FFAAS0414	China	ON427728	ON468453	ON468466	[20]
*M. rufobrunnea*	FFAAS0416, holotype	China	ON427730	ON468455	ON468468	[20]
*M. rubromarginata*	JV362	Denmark	FN394624	KF723705	KF723659	[16,18]
*M. seminau*	ACL136, holotype	Malaysia	KF537250	–	–	[1]
*M. seminau*	ACL308	Malaysia	KF537252	–	–	[1]
*M. shengshanensis*, holotype	FFAAS0424	China	ON427739	ON468464	ON468477	[20]
*M. shengshanensis*	FFAAS0425	China	ON427740	ON468465	ON468478	[20]
*M. sinar*	ACL092	Malaysia	KF537247	–	–	[1]
*M. sinar*	ACL135, holotype	Malaysia	KF537249	–	–	[1]
*M. sinar* var. *tangkaisinar*	ACL307, holotype	Malaysia	KF537251	–	–	[1]
***M. sophiae***	**ACP2157, holotype**	**Mexico**	**OR233606**	**OR233739**	**OR233749**	**This study**
***M. sophiae***	**ACP2161**	**Mexico**	**OR233605**	**–**	**OR233757**	**This study**
*M. subulata*	FFAAS0419	China	ON427735	ON468460	ON468473	[20]
*M. subulata*	FFAAS0423, holotype	China	ON427737	ON468462	ON468475	[20]
*M. yuezhuoi*	FFAAS0344	China	MW581490	MW868166	MW882249	[19]
*M. yuezhuoi*	FFAAS0347	China	MW581493	MW868167	MW882252	[19]

* Correct voucher is LK1191/2002 instead of LK1191/2000 at GenBank.

## Data Availability

The sequences presented in this study are available at https://www.ncbi.nlm.nih.gov/ accessed on 1 July 2023 (see Table 1 for the accession numbers). The alignments and phylogenetic tree files can be requested directly to the authors. All new taxa were registered in the MycoBank (http://www.mycobank.org/ accessed on 1 July 2023).
